# Study of the underlying mechanisms and consequences of pathogenicity differences between two in vitro selected G1-H9N2 clones originating from a single isolate

**DOI:** 10.1186/s13567-019-0635-1

**Published:** 2019-03-01

**Authors:** Giang Thu Nguyen, Fabienne Rauw, Mieke Steensels, Fiona Ingrao, Francesco Bonfante, Irit Davidson, Bénédicte Lambrecht

**Affiliations:** 1 Avian Virology and Immunology Service, National Reference Laboratory for Avian Influenza and Newcastle Disease Virus, Sciensano, Uccle, Brussels Belgium; 2 Istituto Zooprofilattico Sperimentale Delle Venezie, Legnaro, Italy; 3 Division of Avian and Diseases, Kimron Veterinary Institute, Bet Dagan, Israel

## Abstract

The G1-H9N2 avian influenza virus (AIV) has caused significant economic losses in the commercial poultry industry due to reduced egg production and increased mortality. The field observations have shown that H9N2 viruses circulate and naturally mix with other pathogens and these simultaneous infections can exacerbate disease. To avoid an incorrect virus characterization, due to co-infection, isolates were purified by in vitro plaque assays. Two plaque purified G1-H9N2 clones, selected on different cell types, named MDCK-and CEF-clone in regards to the cell culture used, were studied in vivo, revealing two different virulence phenotypes. Subsequently, the underlying mechanisms were studied. Specifically, the phenotypical outcome of SPF bird infection by the two clones resulted in completely different clinical outcomes. These differences in clinical outcome were used to study the factors behind this output in more detail. Further studies demonstrated that the more severe disease outcome associated with the MDCK-clone involves a strong induction of pro-inflammatory cytokines and a lack of type I interferon production, whereas the mild disease outcome associated with the CEF-clone is related to a greater antiviral cytokine response. The immunosuppressive effect of the MDCK-clone on splenocytes was further demonstrated via ChIFN-γ lack production after ex vivo mitogenic stimulation. Genome sequencing of the two clones identified only four amino acid differences including three in the HA sequence (HA-E198A, HA-R234L, HA-E502D-H9 numbering) and one in the NA sequence (NA-V33M). In the present study, valuable insights on the mechanisms responsible for AI pathogenicity and molecular mechanisms of H9N2 infections in chicken were obtained while highlighting the impact of the cells viruses are grown on their virulence.

## Introduction

Avian influenza, commonly known as avian flu or bird flu, is caused by influenza A viruses. Avian influenza viruses (AIVs) are members of the *Orthomyxoviridae* family and they have segmented, negative-sense, single-stranded RNA genomes. Avian influenza infections are some of the most contagious and devastating diseases that currently affect poultry populations. Infections from AIVs can lead to clinical pathologies that range from asymptomatic and mild clinical symptoms to sudden and complete mortality. Influenza A viruses are divided into subtypes based on the expression profile of two glycoproteins on their surface—the hemagglutinin (HA) and the neuraminidase (NA). To date, 18 subtypes of hemagglutinin (H1 to H18) and 11 subtypes of neuraminidase (N1 to N11) have been identified [1]. As a result, many different combinations of HA and NA proteins are possible. The natural reservoir of type A influenza viruses are wild aquatic birds, *Anseriformes* and *Charadriiformes*, such as wild ducks, gulls, and shorebirds [2, 3]. Occasionally, other species such as poultry and mammalian species become infected [4]. AIVs can be classified as low pathogenic (LPAI) or highly pathogenic (HPAI) based on the genetic features of a virus and the severity of disease it causes in chickens. The HPAI has only been associated with some strains of the H5 or H7 subtype [5].

The LPAI H9N2 was first detected in turkeys in the United States in 1966 [6] and distinct lineages have been described in the meantime. A recent study on H9 phylogeny revealed that the panoramic view of the global distribution of H9N2 is more complicated than the separation in North American and Eurasian lineages as thought in the past [7]. To date, four primary lineages, h9.1–h9.4, have been designated to represent its distribution. Lineages h9.1 and h9.2 correspond to viruses which have been isolated in North America in 1966 and in the 1990s, respectively; lineage h9.3 is widely distributed among regions of Asia, Europe, Africa, the Pacific, and North America; and lineage h9.4 has circulated exclusively in Asia with h9.4.1 and h9.4.2 sublineages, comprising the G1-like (h9.4.1.1) and Y280-like (h9.4.2.4) viruses.

The H9 G1-like viruses are the most widespread, with G1-H9N2 infections reported in Asia, the Middle East and North Africa [8, 9]. In the field, G1-H9N2 viruses induce moderate to severe clinical signs in chickens with highly variable rates of mortality (10–60%) and reduced laying rates (14–75%) that lead to economic losses [10, 11]. Numerous in vivo and in vitro studies have been conducted to investigate G1-H9N2 pathogenesis in chickens [12–17]. However, significant contradictions have been observed among the results of these studies and field data. In some experimental studies, H9N2 infections did not induce obvious clinical signs or death and replication of the virus was limited to the upper respiratory tract in specific-pathogen-free (SPF) chickens under laboratory conditions [12–14]. In contrast, other studies reported that H9N2 causes moderate to severe respiratory infections and low mortality in commercial broiler chickens, in parallel with the replication of the virus in multiple tissues [15–17]. Many factors can affect disease outcome. These contradictory results may be explained by pathogenic variability among H9N2 strains, including molecular determinants which have been found to govern the pathogenicity of AIV in chickens [18] or via secondary infections in the field. Indeed, field observations have shown that H9N2 viruses circulate and naturally mix with other pathogens such as *Escherichia coli*, *Mycoplasma gallisepticum*, *Staphylococcus aureus*, *Haemophilus paragallinarum*, *Chlamydia psittaci*, *Ornithobacterium rhinotracheale*, live bronchitis virus vaccine [19]. And these co-infections can exacerbate disease outcome, causing considerable economic losses due to poor weight gain, dramatic drop in egg production and high mortality. In Israel, the common co-circulation of H9N2 and Newcastle disease virus (NDV) since the year 2000 [20] creates complicated scenarios with the reciprocal interference between the two viruses depending on many factors such as intervals of infection, type of virus, dose, virulence and biological properties of viruses, birds species [21]. In fact, H9N2 has been associated with breakthroughs in the NDV mandatory vaccination control program [22]. Ellakany et al. observed that H9N2 infection reduced ND vaccine efficacy [23]. The presence of H9N2 in the host can induce a negative impact on the production of anti-Newcastle disease antibodies (i) either by the viral interference in which the growth of lentogenic Lasota strain—a commonly used as live vaccines against virulent form of NDV—could be suppressed or delayed [24] (ii) or by the immunosuppressive effect in which the immune organs could be damaged or destroyed following H9N2 infection [25, 26]. Furthermore, even in the presence of good herd immunity against NDV, H9N2 is also able to cause a vaccinal break as the simultaneous infection of H9N2 made birds more susceptible to velogenic NDV by lowering the minimum dose required to establish an infection and exacerbating clinical signs [27]. While it is hypothesized that the H9N2 virus acts as an immunosuppressive agent [28], the immunosuppressive effect of Israeli H9N2 and the mechanism behind remain to be fully characterized. To study the mechanisms underlying enhanced pathogenicity of H9N2 AIV and its immunosuppression, two H9N2 clones purified from the same isolate but exhibiting different pathogenicity in chickens were used as appropriate models. The objectives of this study include: (i) assessing the pathogenesis of the two H9N2 clones in SPF chickens (ii) investigating the potential immunosuppressive effects of these H9N2 viruses (iii) exploring the host’s innate immune responses by measuring the expression of different innate immune-related genes (iv) and analysing the genome sequences of both H9N2 clones.

## Materials and methods

### Virus strains

The low pathogenic isolate used in this study was the virus stock A/chicken/Israel/1163/2011 H9N2 LPAI (GenBank accession number: JQ973660.1). This strain was originally isolated from tracheal swabs collected from a 5-week-old broiler flock. The virus culture was amplified in embryonated SPF chicken eggs [29], allantoic fluid stored at −80 °C, and subsequently used as a virus stock. The median 50% egg infectious dose (EID_50_) per mL of this virus was determined by titration on SPF eggs and Reed and Muench calculations [30].

### Virus purification by plaque assay

#### Cell culture

Chicken embryo fibroblast (CEF) cells were prepared from 9-day-old SPF embryonated eggs (Lohmann Valo, Germany) as previously described [31] and were maintained in L15/McCoy medium (Sigma-Aldrich, St. Louis, MO, USA) supplemented with 2% fetal bovine serum (FBS), 50 µg/mL gentamicin and 1 mM glutamine (Thermo Fisher Scientific, Waltham, MA, USA) at 37 °C in 5% CO_2_.

#### Infection

Confluent monolayers (80–90%) of CEF cells were infected with H9N2 virus at varying multiplicity of infection (MOI) (1, 0.1, 0.01, and 0.001) with supplemental tolylsulfonyl phenylalanyl chloromethyl ketone (TPCK)-trypsin (Sigma-Aldrich, St. Louis, MO, USA) at 0.05 µg/mL, in the absence of FBS. After 1 h at 37 °C and 5% CO_2_, the inoculum was removed and cell medium supplemented with antibiotics (50 µg/mL gentamicin, 1 mM glutamine) and 0.05 µg/mL TPCK-trypsin, in the absence of FBS, containing 2% low melting point SeaPlaque™ agarose (Lonza, Basel, Switzerland) replaced the infecting medium. The plates were subsequently incubated upside down at 37 °C in 5% CO_2_ for 3 days.

#### Virus isolation

Virus plaque was formed when a virus particle infected the host cell and inducing cytopathic effects in this and the surrounding cells. Virus plaque was subsequently collected as previously described [32]. Each plaque represented a single pure H9N2 clone. Purified virus was subsequently amplified in SPF embryonated chicken eggs. Egg fluids were collected and titrated (10^−4^ to 10^−9^ dilution) before being aliquoted and stored at −80 °C.

The similar process of plaque purification was performed on MDCK cell culture at Instituto Zooprofilattico Sperimentale delle Venezie, Italy. Two virus clones were purified from two different cell cultures and named CEF-clone and MDCK-clone.

### Experimental design

In vivo experiments were performed in SPF White Leghorn chickens (Lohmann Valo, Germany) under biosecurity level 3 (BSL-3) conditions. The chickens had access to feed and water ad libitum throughout the experiments. Animal experiments were conducted with the authorization and supervision of the Biosafety and Bioethics Committees at Sciensano-Avian Virology and Immunology unit (Brussels, Belgium) according to National and European regulations.

In the first experiment, 4 week-old chickens were randomly divided into two groups, one group (*n* = 43) was infected with the H9N2 CEF-clone via the oculo-nasal route (50 µL intranasal and 50 µL as an eye-drop) at a viral dose of 10^6^ EID_50_ in 100 µL phosphate-buffered saline (PBS) and the other group (*n* = 15) was left untreated and kept separately as non-infected negative controls.

In the second experiment, 4 week-old chickens were randomly divided into two groups, one group (*n* = 37) was infected with the H9N2 MDCK-clone as described above and the other group (*n* = 15) was left untreated and kept separately as non-infected negative controls.

In both experiments, the infected chickens were monitored daily for clinical signs (including conjunctivitis, excessive lacrimation and ruffled feathers) and mortality. The degree of severity of clinical signs was scored as follows: 0 = no clinical signs, 1 = mild to moderate clinical signs, 2 = moderate to severe clinical signs, 3 = dead. Daily clinical score was the average clinical score of the remaining chickens which was calculated from the sum of the individual clinical scores from remaining chickens divided by the number of animals. At 3 and 5 days post-infection (dpi), spleens from infected and non-infected chickens (*n* = 5) were collected for splenocyte isolation and ex vivo mitogenic activation (as described below).

Swabs (tracheal and cloacal) were collected from the infected chickens at 2, 3, 5, 7, 10, 15, and 21 dpi (*n* = 5 in Experiment 1; *n* = 3 in Experiment 2) and then were stored in brain-heart infusion (BHI) broth supplemented with antibiotics (10^7^ U/L penicillin, 2 g/L streptomycin, 1 g/L gentamicin, 0.65 g/L kanamycin) at −80 °C until further analysed.

Tissues (brain, trachea, lung, liver, duodenum, spleen, kidney, and bursa of Fabricius) were resected from infected chickens at 1, 3, 5, 7, 10, 15, 21 dpi and non-infected chickens at 1, 3, 5 dpi. The collected tissues were stored in RNA*later*^®^ solution (Ambion, Applied Biosystems, Carlsbad, CA, USA) and quickly frozen at −80 °C until subjected to RNA extraction for studies of viral presence (*n* = 3) and cytokine expression (*n* = 5).

Blood was collected immediately after the sacrifice of the birds at the end of the experimental period (21 dpi) (*n* = 5 in Experiment 1; *n* = 3 in Experiment 2). Sera were separated by centrifugation at 5000 rpm for about 10 min and were kept at −20 °C until used for the study of seroconversion by hemagglutination inhibition (HI) test.

### RNA extraction and cDNA synthesis

RNA extraction from swabs and tissues (approximately 30 mg) was performed as previously described [33] by using the MagMax AI/ND 96 Viral RNA Isolation kit and the MagMAX-96 Total RNA Isolation kit (Ambion, Applied Biosystems, Carlsbad, CA, USA), respectively, following the manufacturer’s instructions. The purified RNA (100 ng) from the trachea, duodenum and spleen were then reverse-transcribed to cDNA with the GoScript™ Reverse Transcription System (Promega, Madison, WI, USA) for the study of cytokine expression. The cDNA products were stored at −20 °C until they were further analysed.

### Measurement of virus shedding via swabs and tissues

Quantitative real-time reverse transcription (RRT)-PCR targeting of the type A influenza matrix (M) gene was performed using the AgPath-ID One-Step RT-PCR Kit (Ambion, Applied Biosystems, Carlsbad, CA, USA). Primers specific for universal M For/Rev and probe used were previously described by Spackman et al. [34] with a final concentration of 900 nM of each primer (M + 25F and M − 124R) and 400 nM of the TAQMAN^®^-probe in a total reaction volume of 25 µL, containing 2 µL of purified RNA. Amplification and fluorescence detection were performed on LightCycler^®^480 Real-Time PCR system (Roche, Mannheim, Germany) with cycling condition: 50 °C for 30 min and 95 °C for 15 min, followed by 40 cycles of 95 °C 15 s–54 °C 35 s–72 °C 10 s. A crossing-point (Cp) value of 40 was chosen as cut-off and samples with higher Cp were considered as negative. Absolute quantification was done relative to a standard curve based on tenfold dilutions of an in vitro transcribed RNA template. Data are expressed as the number of viral RNA copies (log_10_) per mL for swabs and per 30 mg for tissues. The detection limit was 2log_10_ RNA copies/mL swabs and 2.5log_10_ RNA copies/30 mg tissue.

### Hemagglutinin inhibition (HI) test

HI tests were performed according to the Manual of Diagnostic Tests and Vaccines for Terrestrial Animals [35]. The homologous virus was used as antigen in the HI test. The geometric mean of HI titers was expressed as reciprocal log_2_.

### Measurements of immunosuppression

Splenocytes (*n* = 5) were isolated and then either cultured in RPMI 1640 medium (Invitrogen) in the presence or absence of pokeweed mitogen (PWM) (Sigma-Aldrich, St. Louis, MO, USA), as previously described [36]. PWM is a stimulator of chicken T- and B-cell proliferation and was showed to induce IFN-γ production by splenocytes [36]. T-cell stimulation was measured in supernatants after 48 h at 39 °C using ChIFN-γ-capture specific enzyme-linked immunosorbent assays (ELISAs), as previously described [36]. Cellular responses were expressed as optical density (OD) values. Negative chickens with an OD value < 0.1 for mitogen activation were excluded from further antigen activation analysis. Supernatant of mitogenic activated splenocytes from non-infected chickens as an in-house positive control were used in each plate.

### Measurement of innate immune response-related gene expression

Expression of innate immune response-related genes was obtained by using the Power SYBR^®^ Green RNA-to-CT™ 1-Step kit (Applied Biosystems, Carlsbad, CA, USA) with primers specific for target genes (Table 1) on the LightCycler^®^480 Real-Time PCR system (Roche, Mannheim, Germany), as previously described [37].

**Table 1:** **List of primers used to quantify the relative expression of immune genes by real-time RT-PCR**

	Forward primer	Reverse primer	References
GADPH	5′-GACGTGCAGCAGGAACACTA-3′	5′-TCTCCATGGTGGTGA AGACA-3′	[67]
IL-6	5′-ATCCGGCAGATGGTGATAAA-3′	5′-CCCTCACGGTCTTCTCCATA-3′	[67]
IL-1β	5′-GCTCTACATGTCGTGTGTGATGAG-3′	5′-TGTCGATGTCCCGCATGA-3′	[68]
INF-α	5′-GACATGGCTCCCACACTACC-3′	5′-AGGCGCTGTAATCGTTGTCT-3′	[67]
INF-β	5′-GCCCACACACTCCAAAACACTG-3′	5′-TTGATGCTGAGGTGAGCGTTG-3′	[69]
INF-γ	5′-GTGAAGAAGGTGAAAGATATCATGGA-3′	5′-GCTTTGCGCTGGATTCTCA-3′	[68]
TLR-7	5′-TCTGGACTTCTCTAACAACA-3′	5′-AATCTCATTCTCATTCATCATCA-3′	[70]
TLR-3	5′-GCTATTGAGCAAAGTCGAGA-3′	5′-ACAGGGGGCACTTTACTATT-3′	[70]

Data were normalized to levels of the housekeeping gene, glyceraldehyde-3-phosphate dehydrogenase (*GAPDH*), and then were quantified according to the 2^−∆∆Ct^ method [38]. Changes in gene expression that were detected in the CEF-infected group and in the MDCK-infected group are presented as fold-increases relative to the levels detected in the non-infected group. Samples in which target genes were not detected were excluded from these calculations.

### Whole virus genome Sanger sequencing

RNA was extracted with a HIGH PURE^®^ Viral Nucleic Acid kit (Roche, Mannheim, Germany), according to the manufacturer’s instructions. Reverse transcription (RT) was performed using the Uni12 primer and SuperScript III™ reverse transcriptase (Invitrogen) following the manufacturer’s instructions. RT-PCR was conducted using AccuPrime Taq DNA Polymerase, High Fidelity (Thermo Fisher Scientific, Waltham, MA, USA). All segments were amplified with Hoffman’s primers [20]. The PCR products were purified using the High Pure PCR Product Purification Kit (Roche, Mannheim, Germany) after separation on 1.5% agarose gel. The fragments were sequenced using a BigDye Terminator v3.1 Cycle Sequencing Kit (Applied Biosystems, Carlsbad, CA, USA), following the manufacturer’s instructions. Sequenced fragments were analysed and aligned by using DNAsis Max v 2.05 (Hitachi Software) and a consensus sequence was determined from all replicate sequences.

### Statistical analysis

Analysis of data was performed with Minitab 13 (Minitab Ltd, Coventry, UK) and STATA 10 (Stata Corp LP, TX, USA) statistical programs for Windows 2000. Analyses of innate immune response-related gene expression data were performed with Student’s *t*-test. If normality and homogeneity of variance were not validated, then the non-parametric Wilcoxon-Mann–Whitney’s test was applied. Differences with a *P*-value ≤ 0.05 were considered statistically significant.

## Results

### Pathogenicity in SPF chickens

#### Clinical manifestations

**Figure 1:**
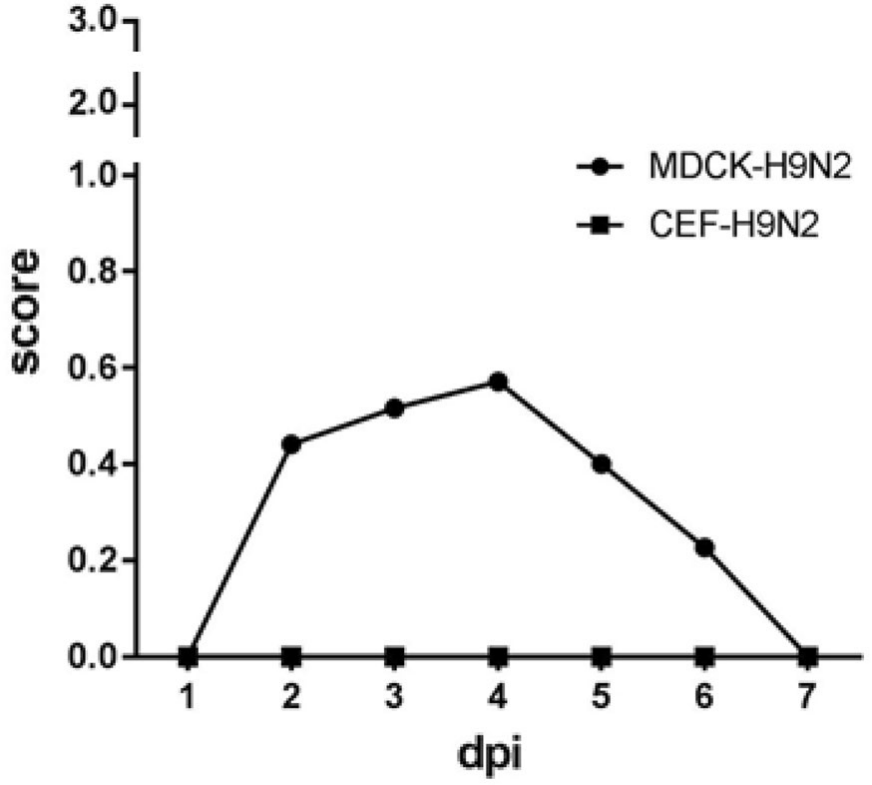
**Course of clinical signs for chickens infected with either CEF-H9N2 or MDCK-H9N2.** Clinical signs were scored as: 0 = no sign, 1 = mild to moderate signs, 2 = moderate to severe signs, 3 = dead. Daily clinical score was calculated from the sum of the individual clinical scores from remaining chickens divided by the number of animals.

Chickens infected with the CEF-clone exhibited no clinical signs of infection. In contrast, the chickens infected with the MDCK-clone exhibited obvious clinical signs which included conjunctivitis, excessive lacrimation and ruffled feathers. These signs were visible from 2 dpi, most remarkable at 4 dpi, and then reduced by 6 dpi (Figure 1). No mortality was recorded in any of the infected groups over the course of the experimental period.

#### Viral dissemination

**Figure 2:**
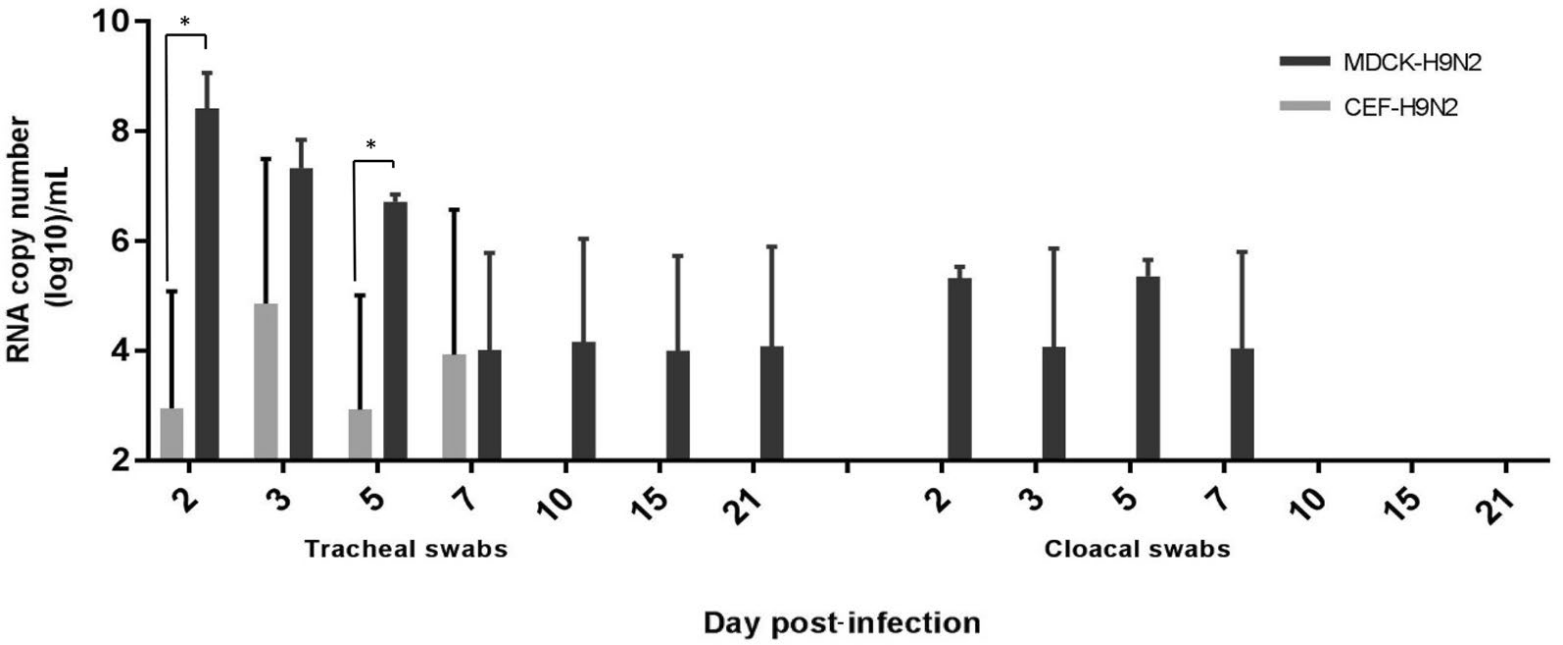
**Viral RNA copy number in swabs collected from chickens infected with either CEF-H9N2 or MDCK-H9N2.** Chickens were infected with two different H9N2 clones and viral excretion was detected in swabs collected at 2, 3, 5, 7, 10, 15, and 21 dpi. The data were expressed as mean ± standard deviation. The limit of detection is 2log_10_ copies/mL. An asterisk indicates statistically significant differences between the two clones (*P* < 0.05).

**Figure 3:**
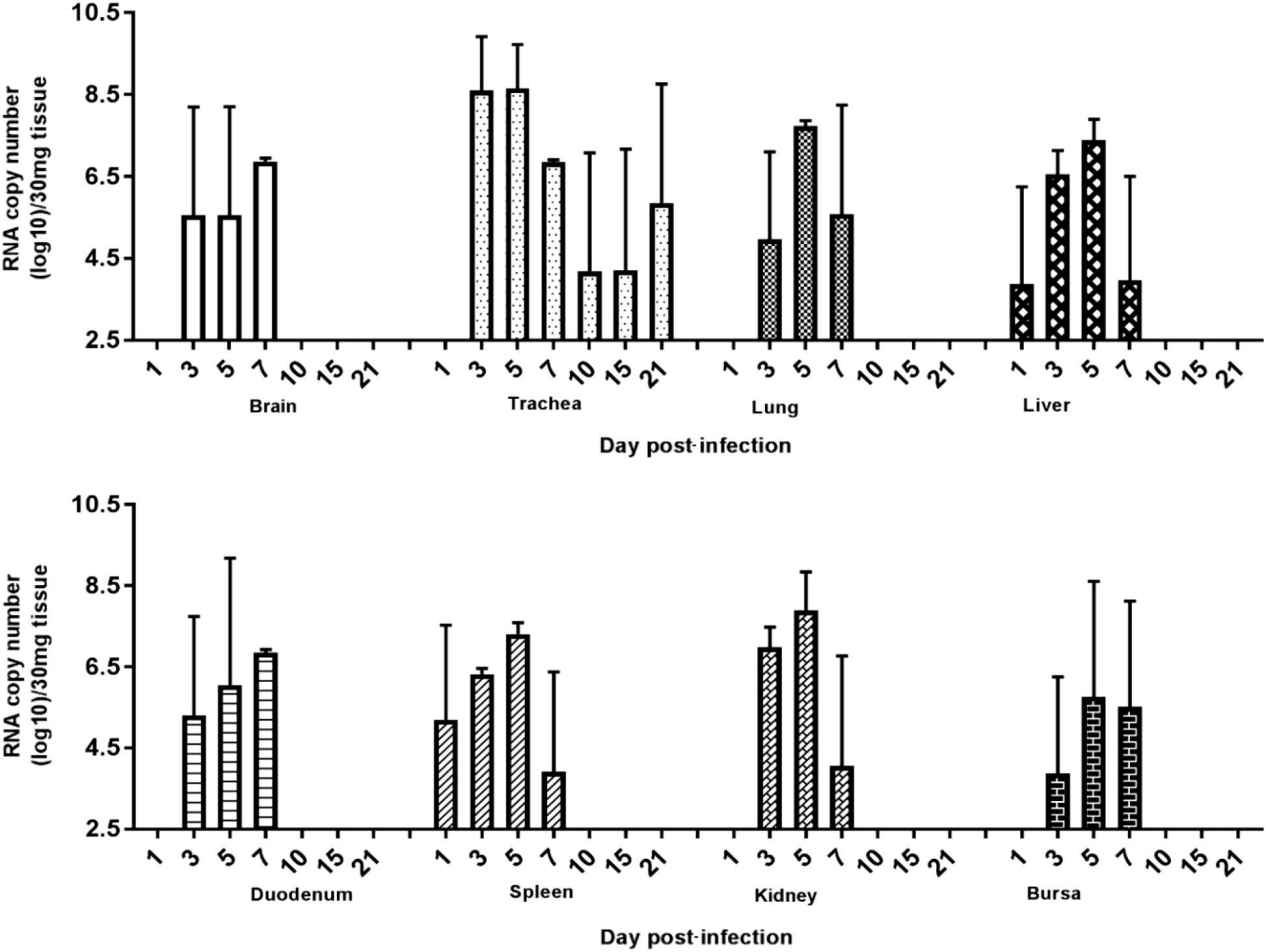
**Viral RNA copy number in various tissues of chickens infected with MDCK-H9N2.** Chickens were infected with MDCK-H9N2 clone and viral excretion in various tissues was subsequently examined at 1, 3, 5, 7, 10, 15, and 21 dpi. The data were expressed as mean ± standard deviation. The limit of detection is 2.5log_10_ copies/30 mg.

Excretion of the CEF-clone was detected in one out of five (1/5) tracheal swabs at 2 dpi (Figure 2). Excretion continued at 3 dpi (3/5) and lasted until 7 dpi (2/5). No viral RNA was detected in any of the cloacal swabs at any sampling point. In none of the examined tissues viral RNA copies of the CEF-clone were detected (data not shown). In contrast, the MDCK-clone exhibited enhanced replication in the chickens with excretions that were detected in both the tracheal and cloacal swabs. High number of viral RNA copies were first detected at 2 dpi (3/3) in the tracheal swabs in this MDCK group (Figure 2). The number of RNA copies in tracheal swabs subsequently decreased although replication of the MDCK-clone was still detected at the end of the experimental period (21 dpi). When compared to the CEF-clone, the MDCK-clone induced significantly higher excretion in tracheal swabs at 2 and 5 dpi. Viral excretion of the MDCK-clone via the cloacal route was also detected at 2 dpi (3/3), at 5 dpi (3/3), and 7 dpi (3/3) (Figure 2). However, from 10 dpi no more viral RNA was detected in cloacal swabs. In addition, the viral RNA was detected in various examined organs from the chickens that were inoculated with the MDCK-clone (Figure 3): in brain, trachea, lung, duodenum, kidney, and bursa of Fabricius tissues, viral RNA copies were detected at 2 dpi, 5 dpi (3/3) and 7 dpi (3/3) while in liver and spleen the viral RNA was detected as early as 1 dpi, 3 dpi and 5 dpi. The viral RNA was undetectable in these organs at 10 dpi.

### Serology

At 21 dpi, the mean log_2_ antibody titers for the CEF- and MDCK-clone-infected groups were 7.2 ± 0.84 (5/5) and 10 ± 0 (3/3), respectively (data not shown).

### Immunosuppression measured by ex vivo mitogenic activation of splenocytes

In the present study, the immunosuppressive effect of the two H9N2 clones was evaluated based on the ability of splenocytes to produce ChIFNγ following ex vivo mitogenic stimulation. PWM was used to activate splenocytes obtained from infected and non-infected chickens at 3 dpi and 5 dpi. ChIFN-γ production measured by ELISA by splenocytes obtained from both CEF- and MDCK-clone infected chickens was downregulated at 3 dpi but only significantly lower from negative group (*P* < 0.05) for MDCK-clone infected chickens (Table 2). At 5 dpi, mitogenic activation was fully restored in CEF-clone infected chickens, while a downregulation was still observed in the MDCK-clone infected birds, although not statistically significant (Table 2).

**Table 2:** **ChIFN-γ production of splenocytes after ex vivo mitogenic activation**

Experiment	Group	ChIFNg production (OD)	
		3 dpi	5 dpi
I	Negative	0.334 ± 0.124	0.683 ± 0.411
	CEF-H9N2 inoculated	0.028 ± 0.002	0.217 ± 0.157
II	Negative	0.489 ± 0.422	0.376 ± 0.256
	MDCK-H9N2 inoculated	0.012 ± 0.001*	0.017 ± 0.002

Splenocytes were stimulated with PWM (10 µg/mL). Forty-eight hours later, supernatants were harvested and ChIFN-γ production was determined by ChIFN-γ capture ELISA. Data represent the mean ± standard deviation of the OD values (*n* = 5).

* Significant differences between naïve and infected chickens (*P* < 0.05).

### Innate immune response

#### Expression of toll-like receptor (TLR) 7 and 3

**Figure 4:**
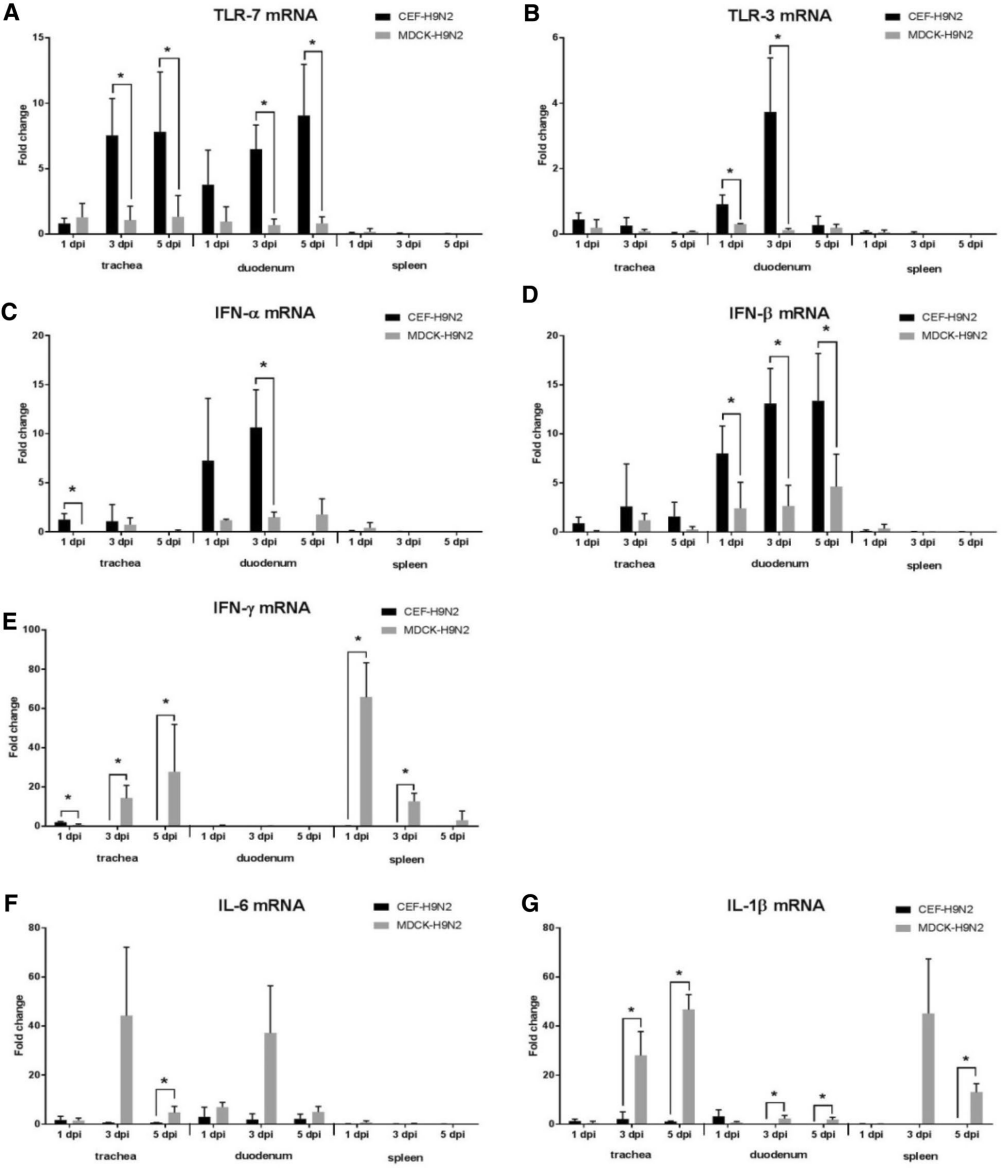
**Innate immune response-related gene expression profiles in chickens infected with either CEF-H9N2 or MDCK-H9N2.** At 1, 3, and 5 dpi, trachea, duodenum, and spleen tissues were resected from chickens infected with CEF-H9N2 or MDCK-H9N2 and mRNA levels of *TLR*-*7* (**A**), *TLR*-*3* (**B**), *IFN*-*α* (**C**), *IFN*-*β* (**D**), *IFN*-*γ* (**E**), *IL*-*6* (**F**), and *IL*-*1β* (**G**) were detected in each by RRT-PCR. Fold-changes in gene expression were calculated relative to the corresponding levels detected in non-challenged chickens. Data were normalized to GAPDH expression, calculated according to the 2^−∆∆Ct^ method [38], and expressed as mean ± standard deviation. An asterisk indicates statistically significant differences between the two clones (*P* < 0.05).

The expression of TLR-7 and TLR-3 was induced in trachea and duodenum by both clones. The chickens infected with CEF-clone expressed significantly higher levels of *TLR*-*7* mRNA in the trachea and duodenum at 3 dpi and 5 dpi (Figure 4A) and higher levels of *TLR*-*3* mRNA in the duodenum at 1 dpi and 3 dpi (Figure 4B). In the spleen, no changes in TLR-7 or TLR-3 expression were measured in either of the infected groups.

#### Expression of antiviral cytokines

In the present study, *IFN*-*α* mRNA was induced rapidly after infection and increased in the trachea and duodenum at 1 dpi and 3 dpi in the chickens infected by the CEF-clone. In contrast, the MDCK-clone induced slightly elevated levels of *IFN*-α mRNA expression in the trachea alone. The difference in *IFN*-*α* mRNA expression was statistically significant in the trachea at 1 dpi and in the duodenum at 3 dpi (Figure 4C). Similarly, the level of *IFN*-*β* mRNA was comparatively up-regulated in the chickens infected with CEF-clone in the trachea and duodenum (Figure 4D). However, the difference in the expression levels of IFN-β was only significant in the duodenum at all of the tested time points. In contrast to type I IFNs, significantly higher levels of *IFN*-*γ* mRNA was induced by MDCK-clone in the trachea at 3 dpi and in the spleen at 1 dpi and 3 dpi (Figure 4E).

#### Expression of pro-inflammatory cytokines

*IL*-*6* mRNA was induced to a greater extent by the MDCK-clone in the trachea and duodenum at all of the tested time points, while a significant difference was detected in the trachea at 5 dpi (Figure 4F). Similarly, *IL*-*1β* mRNA was induced to a significantly greater level by the MDCK-clone compared to the CEF-clone in all of the tissues examined at 3 dpi and 5 dpi (Figure 4G).

### Genome sequencing

A total of four amino acid substitutions were detected in the full genome consensus sequence between the CEF- and MDCK-clone. Three of these substitutions involved the HA gene: E198A, R234L, and E502D in the CEF-compared to the MDCK-clone. In the NA sequence, a single substitution was detected at residue 33, with a valine to methionine shift between the CEF- and MDCK-clone (V33M).

## Discussion

In this study, two clones from a single G1-H9N2 field isolate, obtained via plaque purification on two different cell cultures, were evaluated for their difference in pathogenicity. These two purified clones, the CEF and MDCK-clones demonstrated significant differences in pathogenicity in SPF chickens, allowing studying the underlying mechanism(s) mediating the H9N2 virulence in chicken. Under experimental conditions, the CEF-clone was avirulent in SPF chicken with a local respiratory replication detected in tracheal swabs. In contrast, the MDCK-clone exhibited systemic replication which has been described for other H9N2 strains, such as A/Chicken/Saudi Arabia/SP02525/3AAV/2000 and A/Chicken/HS/K5/01 (H9N2) with viral RNA detection in multiple tissues including brain, trachea, lung, ileum, liver, kidney and spleen [39, 40]. However, the information on the potential transmissibility of two H9N2 clones was lacking as sentinel animals were not included in this study. In addition, the work presented here demonstrated that the MDCK-clone could play a role as a stronger immunosuppressive agent. Indeed, in splenocytes from chickens infected with MDCK-clone, an absence of IFN-γ production after ex vivo mitogenic activation confirmed the presence of unresponsive T lymphocytes. Influenza A viruses can cause immunosuppression in chickens by lysing or functionally impairing lymphocytes [41]. Some H9N2 strains have been demonstrated to be lymphotropic and cause severe immunosuppression in chickens by this mechanism [25]. Here, a causal link between the systemic replication of MDCK-clone and its immunosuppressive effect is suggested. However, further investigations are needed to confirm the direct effect of MDCK-clone on lymphocytes by a set of in vitro experiments and flow cytometry.

As pathogenesis and immunosuppression do not only rely on the replicative ability of influenza viruses alone but also on the host’s immune response, early immune responses following either H9N2 clone infection were characterized. Host innate immunity mediates the first line of defense towards influenza infection and TLR-7 and TLR-3 are the main sensors for influenza viruses, responsible for triggering the host’s innate immune responses in chickens [42]. We observed that an avirulent CEF-clone triggered the expression of these two TLRs, whereas the MDCK-clone did not mediate a TLR-7 and TLR-3 response. Interestingly, these results are similar with previous findings that levels of *TLR*-*7* and *TLR*-*3* mRNA are barely up-regulated in response to highly pathogenic H7 infections, while rapid upregulation occurs following a lower pathogenic H7 infection [43]. After influenza virus components being sensed, type I IFNs are produced by different innate immune cells and paramount in the defense against influenza infection with evidence that a greater and longer response of IFN-α was shown to be a crucial factor in host’s strategy against avian influenza [44]. In the present study, the elevated response of type I IFNs in chickens infected with CEF-clone may reflect the effort of the host’s immune system to achieve viral clearance, conversely, the absence of type I IFNs following the infection with MDCK-clone could be associated with the presence of viral mRNA in different organs and prolonged viral shedding in the trachea. The latter case may be a reflection of NS1 interference—a known IFN antagonist [45]. Interestingly, F103L mutation which is related to increased interferon antagonism for avian influenza virus [46] was found in NS1 segment of both clones. The deficient expression of type I IFN following MDCK-clone infection coincided with a high viral RNA load, speculating that the assumed high levels of NS1 viral protein could explain this deficiency by the immunomodulatory capacity described for this protein. The principle of NS1 antagonism in type I IFN induction cascade includes (i) sequestering dsRNA away from host sensors (ii) targeting TRIM25 to inhibit RIG-I activation (iii) interfering the processing and nuclear export of cellular mRNAs, thus limiting type I IFNs expression post-transcriptionally [47]. In addition, the CEF- and MDCK-clone also strikingly differed in Il-6 and Il-1β inductions. These pro-inflammatory cytokines are considered to be the main mediators of inflammation and their expression levels directly correlate with viral replication and the respiratory and systemic symptoms of influenza disease [48]. The expression levels of pro-inflammatory cytokines have been found to be higher in chickens infected with HPAI compared to LPAI [49]. In the present study, the intense expression of these pro-inflammatory cytokine genes by MDCK-clone in the trachea and spleen suggests that its tropism is associated with the upper respiratory tract and lymphoid organs. In contrast, the locally deficient inflammatory response by the CEF-clone may be due to its replicative inability in these tissues (trachea, duodenum, and spleen).

Another dominant difference in response to CEF-clone and MDCK-clone is the expression of IFN-γ. In response to MDCK-clone infection, IFN-γ was significantly elevated in trachea and spleen. Remarkably, the immunosuppression observed on the corresponding splenocytes positively correlated with induction of IFN-γ in the spleen in vivo, suggesting the pivotal role of IFN-γ on the higher and longer immunosuppressive effect induced by MDCK-clone. This view is supported by the previous insights regarding infectious bursal disease (IBDV) infection [50]. The immunosuppressive effect of H9N2 could elucidate its exacerbating role in numerous co-infection cases noticed in the field [51–53] and under experimental conditions [27]. However, the duration and severity of immunosuppression depend on infection dose and age and breed of infected chickens [54, 55].

These data also emphasize that host cells from which the purified clones were obtained can impact their viral pathogenesis. Both CEF and MDCK are widely used cell-cultures as a substrate for the propagation of influenza A viruses [56]. As the AIVs belong to a RNA virus family, they have been shown to have a stable complex distribution of mutant genomes in a population due to error-prone replication, described as quasispecies [57]. The heterogeneity in an influenza virus would persist in one host where variants have equal growth potential but would also be destroyed when introduced into a new host. As a result, when the stock virus is seeded in different cell cultures, different subpopulations may be selected depending on the best host-adaptation condition. The generation of distinct H9N2 clones after a single round of purification on different cell cultures suggests the existence and selection of virus quasispecies with variant virulence in the original H9N2 stock. However, as no next generation sequencing was performed, the proportion of the two cloned variants as quasispecies populations in the original virus stock could not be determined. It should also be pointed out that the presence as quasispecies in the original isolate is not the only explanation for the outcome of two distinct clones from a single field isolate. Also, post-translational modifications, depending on the cell-culture systems used for viral purification, may affect the virulence of the cloned viruses. It was reported that the cells in which the virus was grown, can affect the binding properties of the influenza A hemagglutinin by adding differently structured oligosaccharides [58–60]. Receptor distribution analysis demonstrated a high prevalence of SA 2,3-Gal linkages in CEF cells that validates the high replication of avian influenza viruses. Whereas, MDCK cells express a high amount of both SA α2,6-Gal and α 2,3-Gal linkages on their surface supporting the growth of a broader range of influenza A viruses [61]. In this context, the unique ability of some H9N2 viruses to bind both SA α2,6-Gal and α 2,3-Gal linkages [62, 63] is of importance to explain the multi-tropism of the MDCK-clone G1-H9N2. These hypotheses would merit more in-depth investigations, as the propagation in kidney cell cultures resulted in increased virulence of the H9N2 corresponding with the observation that the infections with some LPAI were nephrotropic during the simulated systemic infection of chickens [64–66]. The biological properties of two H9N2 clones should be explored through the analysis of the plaque morphology, growth kinetics, and receptor binding profiles. Finally, molecular changes such as amino acid substitutions are responsible for phenotypical changes such as virulence-shifts. Strikingly, comparison of the genomic information of the two selected clones revealed only four substitutions, including the residues 198, 234 (equivalent to residue 226 in H3 numbering [8]) and 502 of the HA protein, and residue 33 of the NA protein. All, a combination of some or just one of these substitutions might be responsible for the detected different phenotype in chickens. Residues 198 and 234 are located in the receptor-binding pocket of HA and directly involved in the binding of SA receptors. Any changes here would be predicted to affect linkage specificity and strength. However, no virulence markers have previously been associated with these positions in H9N2. The two other residues in HA and NA are not associated with any functional regions of these glycoproteins and the detected mutations HA-D502E and NA-V33 M have so far not been described as virulence marker.

To our knowledge, this study is the first description of how the purification of an H9N2 isolate on different cell cultures results in the generation of two clones with distinct pathogenic characteristics, induction of different immune profiles and distinct immunosuppression profiles in SPF chickens. Overall, these results suggest that deficient TLR-7 and type I IFNs expression mediates the pathogenicity profile of H9N2 in chickens. In addition, only four mutations that differentiate the two clones. Further research is required to investigate the function of genetic mutations responsible for the major phenotypical differences observed between these two clones by using reverse genetics approach. A full-length cDNA copy of genome from avirulent H9N2 (CEF-clone) could be used as a backbone in which each mutation or a combination of mutations would be introduced and the phenotype of recombinant viruses could be investigated. Furthermore, the quasispecies of the H9N2 original isolate and clones will need to be determined by NGS approaches. Finally, any post-translational modifications that might have occurred will be investigated.

## Abbreviations

AIV: avian influenza virus
BHI: brain–heart infusion
BSL-3: biosecurity level 3
CEF: chicken embryo fibroblast
DMEM: Dulbecco’s modified Eagle’s medium
(c)DNA: (complementary)deoxyribonucleic acid
EID_50_: 50% egg infectious dose
dpi: days post-infection
ELISA: enzyme-linked immunosorbent assay
FBS: fetal bovine serum
GAPDH: glyceraldehyde 3-phosphate dehydrogenase
HA: hemagglutinin
HI: hemagglutinin inhibition
HPAI: highly pathogenic avian influenza
IBDV: infectious bursal disease virus
(ch)IFN: (chicken)interferon
IL: interleukin
LPAI: low pathogenic avian influenza
MDCK: Madin–Darby canine kidney
NA: neuraminidase
(v)NDV: (virulent)Newcastle disease virus
NGS: new generation sequencing
OD: optical density
PBS: phosphate-buffered saline
PWM: pokeweed mitogen
(m)RNA: (messenger)ribonucleic acid
RRT-PCR: real-time reverse transcription polymerase chain reaction
SA: sialic acid
SPF: specific-pathogen-free
TPCK: tolylsulfonyl phenylalanyl chloromethyl ketone
TLR: toll-like receptor
